# Brain injury-associated biomarkers of TGF-beta1, S100B, GFAP, NF-L, tTG, AbetaPP, and tau were concomitantly enhanced and the UPS was impaired during acute brain injury caused by *Toxocara canis *in mice

**DOI:** 10.1186/1471-2334-8-84

**Published:** 2008-06-24

**Authors:** Chien-Wei Liao, Chia-Kwung Fan, Ting-Chang Kao, Dar-Der Ji, Kua-Eyre Su, Yun-Ho Lin, Wen-Long Cho

**Affiliations:** 1 Institute of Tropical Medicine, National Yang-Ming University School of Medicine, 155 Li-Nong St., Sec. 2, Taipei 112, Taiwan; 2 Department of Parasitology, Taipei Medical University College of Medicine, 250 Wu-Hsing St., Taipei 110, Taiwan; 3 Graduate Institute of Medical Sciences, Taipei Medical University College of Medicine, 250 Wu-Hsing St., Taipei 110, Taiwan; 4 Laboratory of Parasitic Diseases, Center for Diseases Control, Department of Health, 161 Kun-Yang St., Taipei 100, Taiwan; 5 Department of Parasitology, National Taiwan University College of Medicine, 1 Jen-Ai Rd., Sec. 1, Taipei 100, Taiwan; 6 Department of Pathology, Taipei Medical University College of Medicine, 250 Wu-Hsing St., Taipei 110, Taiwan

## Abstract

**Background:**

Because the outcomes and sequelae after different types of brain injury (BI) are variable and difficult to predict, investigations on whether enhanced expressions of BI-associated biomarkers (BIABs), including transforming growth factor β1 (TGF-β1), S100B, glial fibrillary acidic protein (GFAP), neurofilament light chain (NF-L), tissue transglutaminases (tTGs), β-amyloid precursor proteins (AβPP), and tau are present as well as whether impairment of the ubiquitin-proteasome system (UPS) is present have been widely used to help delineate pathophysiological mechanisms in various BIs. Larvae of *Toxocara canis *can invade the brain and cause BI in humans and mice, leading to cerebral toxocariasis (CT). Because the parasitic burden is light in CT, it may be too cryptic to be detected in humans, making it difficult to clearly understand the pathogenesis of subtle BI in CT. Since the pathogenesis of murine toxocariasis is very similar to that in humans, it appears appropriate to use a murine model to investigate the pathogenesis of CT.

**Methods:**

BIAB expressions and UPS function in the brains of mice inoculated with a single dose of 250 *T. canis *embryonated eggs was investigated from 3 days (dpi) to 8 weeks post-infection (wpi) by Western blotting and RT-PCR.

**Results:**

Results revealed that at 4 and 8 wpi, *T. canis *larvae were found to have invaded areas around the choroid plexus but without eliciting leukocyte infiltration in brains of infected mice; nevertheless, astrogliosis, an indicator of BI, with 78.9~142.0-fold increases in GFAP expression was present. Meanwhile, markedly increased levels of other BIAB proteins including TGF-β1, S100B, NF-L, tTG, AβPP, and tau, with increases ranging 2.0~12.0-fold were found, although their corresponding mRNA expressions were not found to be present at 8 wpi. Concomitantly, UPS impairment was evidenced by the overexpression of conjugated ubiquitin and ubiquitin in the brain.

**Conclusion:**

Further studies are needed to determine whether there is an increased risk of CT progression into neurodegenerative disease because neurodegeneration-associated AβPP and phosphorylated tau emerged in the brain.

## Background

Brain injury (BI) caused by any number of physical, chemical, or biological insults can have disabling or even fatal consequences [1]. Because the outcomes and sequelae resulting from different types of BI vary and are difficult to predict, BI-associated biomarkers (BIABs) are used to help delineate pathophysiological mechanisms, and predict and monitor neurological outcomes. Some BIABs that have been used are cytokines such as transforming growth factor-β1 (TGF-β1) [2] and S100B [3], glial proteins such as glial fibrillary acidic protein (GFAP) [4] and neurofilament light chain (NF-L) [5], and enzymes such as tissue transglutaminases (tTGs) [6], β-amyloid precursor proteins (AβPPs) [7], tau [8], and ubiquitin [9]. Many, if not all, of these injury-associated factors have the capacity to detrimentally affect the central nervous system (CNS).

TGF-β1 is involved in regulating the brain's response to inflammation and injury. Increased levels of TGF-β1 have been correlated with deposition of scar materials after traumatic CNS injury, and overexpression of TGF-β1 in the CNS might lead to reduced microglial activation and reduced induction of proinflammatory chemokines after severe hypoxic-ischemic injury [10]. The S100B protein, abundant in glial cells of the CNS, predominately in astrocytes, belongs to a multigenic family of calcium-binding S100 proteins. There is substantial evidence that it also exerts a neuropathological influence on the CNS [3].

Astrocytes are supportive structural elements of the nervous system. They play active roles in normal brain physiology and in certain pathological conditions. Reactive astrocytes with increased expression of GFAP are commonly found in cerebral infarction and many areas of brain damage [11]. NF-L is a major component of neuron intermediate filaments, is known to contribute to the rigidity and tensile strength of axons and dendrites, and may play a role in intracellular transport. It has often been found in excess in axons after diffuse brain injury and in abnormal amounts in Alzheimer's disease (AD) [12].

tTG, a Ca^2+^-dependent enzyme, can catalyze the incorporation of a polyamine into polypeptide-bound glutamine leading to the formation of a (γ-glutamyl) polyamine bond resistant to proteolytic cleavage, and may serve not only to stabilize proteins against degradation, but may also alter their functions. Elevated expression of tTG has been observed after traumatic BI (TBI) [6] and cerebral ischemic injury [13]. AβPPs, produced by different cell types including the endothelium, glia, and neurons, are large transmembrane proteins that are thought to play roles in intra- and interneuronal signaling, synaptic transmission, neural growth, as well as plasticity, learning, and memory. AβPPs have been widely detected in a variety of CNS injuries, including brain ischemia and trauma [7], and when aberrantly processed or overproduced, can lead to neurotoxic β-amyloid (Aβ) protein production [14].

The normal function of the tau protein, an important structural element in neuronal cells, is to assemble and stabilize microtubules, but in certain BIs (e.g., TBI or AD), it is redistributed to cell bodies where it accumulates and forms insoluble, fibrillary deposits [15]. The ubiquitin-proteasome system (UPS) functions in cellular quality control by degrading misfolded, unassembled, or damaged proteins that could otherwise form potentially toxic aggregates. Ubiquitin is a small and highly conserved protein found in all eukaryotic cells. One of its major functions is to act as a proteasome pathway, wherein ubiquitination serves as a signal for the target protein to be degraded. The presence of elevated ubiquitin conjugates associated with intracellular deposits of aggregated protein in diseased neurons in nearly all sporadic and hereditary neurodegenerative diseases has long suggested a linkage between UPS dysfunction and pathogenesis, e.g., TBI [9] and AD [16].

*Toxocara canis *is an intestinal nematode of canines, and its embryonated eggs are infectious to both final and paratenic hosts including humans and rodents. *Toxocara canis *larvae, measuring 357~414 μm in length × 13~17 μm in width [17], may invade the brains of paratenic hosts and cause BI, which then may result in cerebral toxocariasis (CT) [18]. However, its effects on the brain are likely to be too cryptic to be clinically detected in humans with CT because the parasitic burden is light; thus, the neuropathogenesis and sequelae of subtle BI in CT remains largely unclear [19-21]. Since CT in humans and mice share similar pathologies involving *T. canis *larvae and migratory pathways [18], it may be possible to detect BIAB and UPS function in a murine model as a means of investigating the pathogenesis of CT.

In this study, we investigated the pathogenesis of CT and the extent of neural damage caused by *T. canis *larvae by measuring levels of BI-associated indicator molecules of TGF-β1, S100B, GFAP, NF-L, tTG, AβPP, and tau as well as UPS function by assessing ubiquitin expression in infected mice brains.

## Methods

### Parasites and the experimental protocols used to induce CT

*Toxocara canis *eggs were obtained from adult female worms, and embryonated eggs were prepared as previously described [22]. Female ICR mice aged 6~8 weeks were obtained from the Center for Experimental Animals, Academia Sinica, Taipei, Taiwan. Mice were housed in the animal facility of Taipei Medical University and maintained on commercial pellet food and water *ad libitum*. The viability of *T. canis *embryonated eggs was assessed by the light stimulation method immediately before use. Each mouse was infected by oral intubation with about 250 embryonated eggs in 100 μl of water [22].

**Figure 1:**
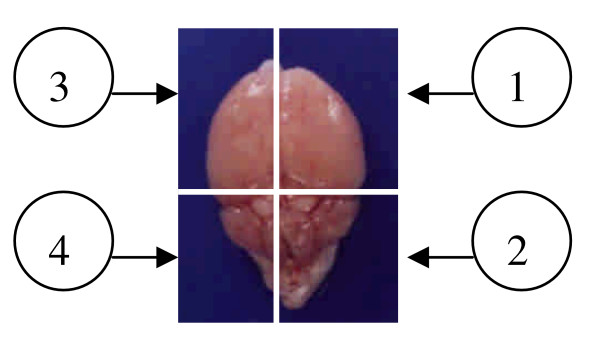
**The relative locations of a mouse brain used for various assays in this study.** 1, the first part for pathological study; 2, the second for larval recovery study; 3, the third for Western blotting; and 4), the fourth for RT-PCR analysis.

Infected mice were deeply anaesthetized with ether and killed by heart puncture on day 3 and at weeks 1, 4, and 8 post-infection (dpi or wpi). On each date, three infected mice and two age-matched uninfected mice were killed, and their brains were excised for further experimentation. As shown in Figure 1, each brain was divided into four parts. The first part of the brain was processed for histological and immunohistochemical studies, and the second part underwent acid-pepsin digestion for larval recovery. The third and fourth parts were processed for Western blotting and reverse-transcription polymerase chain reaction (RT-PCR), respectively (Fig. 1). All animal experiments were carried out in accordance with institutional *Policies and Guidelines for the Care and Use of Laboratory Animals*, *Taipei Medical University *and all effort was made to minimize animal suffering.

### Toxocara canis larval invasion assessed by a larval recovery study

Whether the larvae had successfully invaded the brain was confirmed using a previously described method [23]. Briefly, a brain specimen from each infected mouse was individually ground in a Waring blender (Tatung, Taipei, Taiwan) and then digested in 50 ml of a pepsin/HCl solution (pH 1~2, 10,000 IU, Sigma, Steinheim, Germany) for 3 h at 37°C. Water (50 ml) was added, the mixture was centrifuged (at 250 *g *for 10 min), and the larvae in the sediment were counted in a Petri dish placed under an inverted microscope (Olympus, Tokyo, Japan) at 100× magnification.

### Cerebral injury assessed by pathological changes and astrogliosis detected by the expression of GFAP

A brain specimen from each mouse was separately fixed in 10% neutral buffered formalin for at least 24 h and embedded in paraffin for pathological studies and immunohistochemical detection of GFAP. Five-micrometer brain sections were processed using standard procedures and stained with hematoxylin-eosin (H&E) for the histological study. For the immunohistochemical studies, brain sections from infected and uninfected mice were deparaffinized and rehydrated using descending ethanol gradients before further processing. Immunohistochemical detection of GFAP was performed as described by Balasingam et al. [24]. Briefly, endogenous peroxidase activity was blocked by 3% hydrogen peroxide (Merck, Taufkirchen, Germany). An avidin/biotin blocking kit (SP2001, Vector, Burlingame, CA, USA) was used to block the endogenous avidin/biotin activity to reduce background staining. To eliminate non-specific staining, Fc receptors were blocked with diluted normal goat serum (X0907, Dako, Carpentaria, CA, USA) for 30 min at room temperature in a humid chamber. Sections were then incubated for at least 12 h at 4°C with rabbit anti-mouse GFAP polyclonal antibodies (cat. no. RB-087, Neomarkers, Fremont, CA, USA) diluted in phosphate-buffered saline (PBS) at 1: 160. Sections were then washed with 0.05% Tween 20 Tris-HCl buffer three times for 5 min each. Immunohistochemical detection kits (K4003, Dako) were used to detect GFAP-expressing cells by incubation with horseradish peroxidase-conjugated goat anti-rabbit antibodies for 40 min at room temperature. The presence of peroxidase was detected using chromogen 3,3-diaminobenzidine (DAB) (K3468, Dako). Sections were counterstained with Gill's hematoxylin (H3401, Vector), dehydrated, and mounted in mounting medium (H5000, Neomarkers). In order to confirm the results of the staining, a normal mouse brain section was used as a positive control. Specificity was ascertained by treating positive control sections as described above but omitting the primary antibodies. In addition, for negative controls, we used normal mouse sections subjected to normal rabbit serum as the primary antibody.

### Expressions of TGF-β1, S100B, GFAP, NF-L, tTG, AβPP, Aβ, total (t) and phosphorylated (p) tau proteins, and UPS function as assessed by Western blotting and ELISA

Western blotting was performed as described by Ueberham et al. [25] with modifications. Briefly, brain specimens from infected and uninfected mice were homogenized and lysed in radioimmunoprecipitation assay buffer containing a protease inhibitor cocktail (Sigma, Saint Louis, MO, USA) on ice for at least 1 h, after which the proteins were harvested by centrifugation at 10,000 *g *at 4°C for 10 min, and stored at -80°C for further studies. Before being loaded onto the gel, the protein extracts were boiled for 5 min. In each lane, 50 μg of proteins in loading buffer was boiled and then electrophoresed in a 6%~18% SDS/PAGE mini gel. The proteins were then electrically transferred onto a Hybond-P polyvinylidene fluoride membrane for 2 h. Membranes were blocked in 10% PBS/skimmed milk, and then mouse anti-TGF-β1 monoclonal antibodies (mAbs) (cat. no. T0438, Sigma, USA), mouse anti-S100B mAbs (cat. no. S2532, Sigma), mouse anti-tau mAbs (cat. no. T9450, Sigma), mouse anti-β-actin mAbs (cat. no. A5441, Sigma), goat anti-tTG polyclonal antibodies (pAbs) (cat. no. T7066, Sigma), goat anti-NF-L pAbs (cat. no. sc12980, Santa Cruz Biotechnology, Santa Cruz, CA, US), rabbit anti-GFAP pAbs (cat. no. RB087A1, NeoMarker, Fremont, CA, US), mouse anti-AβPPs mAbs (cat. no. Mab348, Chemicon, Billerica, MA, US), mouse anti-Aβ mAbs (cat. no. AB5078P, Chemicon), or mouse anti-ubiquitin mAbs (cat. no. MAB1510, Chemicon) were added for 2 h at 37°C. After several washes with PBS-Tween 20, a peroxidase-conjugated secondary antibody was added, and the membrane was hybridized at 37°C for 30 min. Immunoreactions were detected with a Western Lightning^® ^kit (Perkin Elmer Life Sciences, Boston, MA, USA). The secondary antibodies, rabbit anti-mouse immunoglobulin G (IgG), goat anti-rabbit IgG (Sigma), and donkey anti-goat IgG (Santa Cruz), were used at 1: 10,000 dilutions. The optical density of the immunoreactive band was measured with a Typhoon 9000D cabinet equipped with ImageQuant software (GE Life Sciences, Fairfield, CT, US). Relative amounts of the targeted proteins were expressed as optical density (OD) relative to that of the control group of uninfected mice. If the p-tau was undetectable by Western blotting, any p-tau proteins in the brains were subjected to further quantitative analysis using an enzyme-linked immunosorbent assay (ELISA) kit (Sigma), which has the ability to detect a minimal dose of p-tau of > 7.4 pg/ml.

### Expressions of TGF-β1, S100B, GFAP, NF-L, tTG, and AβPP mRNA as assessed by RT-PCR

Total RNA was isolated from murine brains using the GenElute™ Mammalian Total RNA Miniprep Kit (Sigma) according to manufacturer's instructions. RT-PCR was performed using a JumpStart™ RED HT RT-PCR Kit (Sigma). One microgram of total RNA was reverse-transcribed using oligo (dT)23 as the primer and enhanced avian myeloblastosis virus reverse transcriptase (eAMV-RT) (Sigma) in a 20-μl reaction mixture. The resulting complementary (c)DNA was amplified using JumpStart REDTaq^® ^DNA polymerase. The primers are indicated in Table 1. Amplified products were resolved on 1.5% agarose gels containing ethidium bromide and visualized in an UV-transilluminator. The optical density of bands was determined with a Typhoon 9000D series (GE Life Sciences). Relative amounts of targeted genes were expressed as an optical density relative to that of GAPDH. Three independent cDNA preparations were analyzed in each experiment.

**Table 1:** Primers of biomarkers, and their source, PCR conditions, and expected size.

Targets	Primer sequence 5'-3'	Annealing temperature	Cycle number	Expected size (bp)	Reference
TGF-β1	sens-AGACGGAATACAGGGCTT TCGATT CA	55°C	35	492	[73]
	antisens-CTTGGGCTTGCGACCCAGTAGTA				
GFAP	sens-GAATGGCCACTAAGGCAGTC	58°C	35	400	[74]
	antisens-TGCACTCCCTCTCTCCTGTT				
S100B	sens-GACTCCAGCAGCAAAGGTGAC	58°C	35	211	[75]
	antisens-CATCTTCGTCCAGCGTCTCCA				
tTG	sens-AGGCCAACCACCTGAACAAA	60°C	35	475	[76]
	antisens-CATACAGGGGATCGGAAAGT				
NF-L	sens-AGCAGAATGCAGACATTAGCG CC	57°C	38	200	[77]
	antisens-TGGTCTCTTCGCCTTCC AAGA GT				
AβPP69	sens-GCACTAACTTGCACGACTAT GGCATGCTGCTGCCCTG	70°C	35	401	
	antisens-GCTGGCTGCCGTCGTGG GAACTCGGACTACCTCCTCCAC A				
AβPP75 1	sens-CTACCACTGAGTCTGTGGAG	64°C	45	222	[50]
	antisens-GCTGGCTGCCGTCGTGG GAAACACGCTGCCACACACCGC C				
AβPP77 0	sens-CTACCACTGAGTCTGTGGAG	64°C	45	242	
	antisens-CTTGAGTAAACTTTGGGA TGACACGCTGCCACACACCGCC				
GAPDH	sens-ACCACAGTCCATGCCATCAC	60°C	27	452	[75]
	antisens-TCCACCACCCTGTTGCTG TA				

### Data analysis

All data of duplicate tests performed in ELISA, Western blotting, and RT-PCR were reported as the mean OD ± standard deviation (SD), and the statistical difference between the experimental group of infected mice and control group of uninfected mice could be calculated by the mean OD values obtained from ELISA, Western blotting, or RT-PCR from each experimental group of 3 mice. Those that were greater than or equal to the mean OD value plus 2, 3, or 4 SDs of the negative control group of 8 uninfected mice were considered to be statistically significant (*P *< 0.05, *P *< 0.01, or *P *< 0.001, respectively), according to a method suggested by Richardson et al. [26] and Tijssen [27].

## Results

### Number of *T. canis *larvae accumulating in the brain increased with time in experimental CT

**Figure 2:**
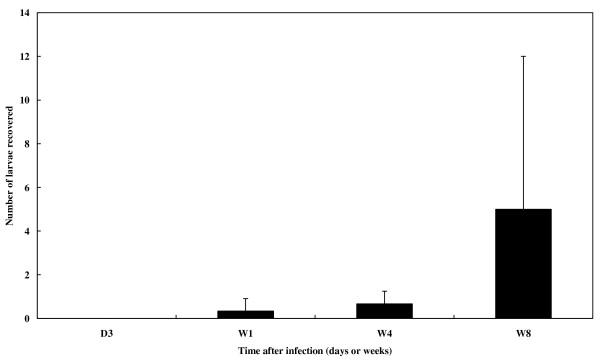
**Recovery numbers of larvae from the brains of *T. canis *infected mice.** Larval numbers are presented as the mean + 1 standard deviation (S.D.) of larvae harvested from the brain of mice infected with embryonated eggs, from 3 days to 8 weeks post infection (dpi or wpi).

No larvae were recovered in brains at 3 dpi. The mean (± SD) number of *T. canis *larvae recovered at 1 wpi was 0.33 0.58, which gradually increased to 0.67 0.58 at 4 wpi. The count had increased to 5.0 7.0 by 8 wpi (Fig. 2). All of the recovered larvae were viable as judged by their motility.

### Areas around the choroid plexus invaded by *T. canis *larvae showed cerebral injury as evidenced by apparent astrogliosis with increased expression of GFAP

**Figure 3:**
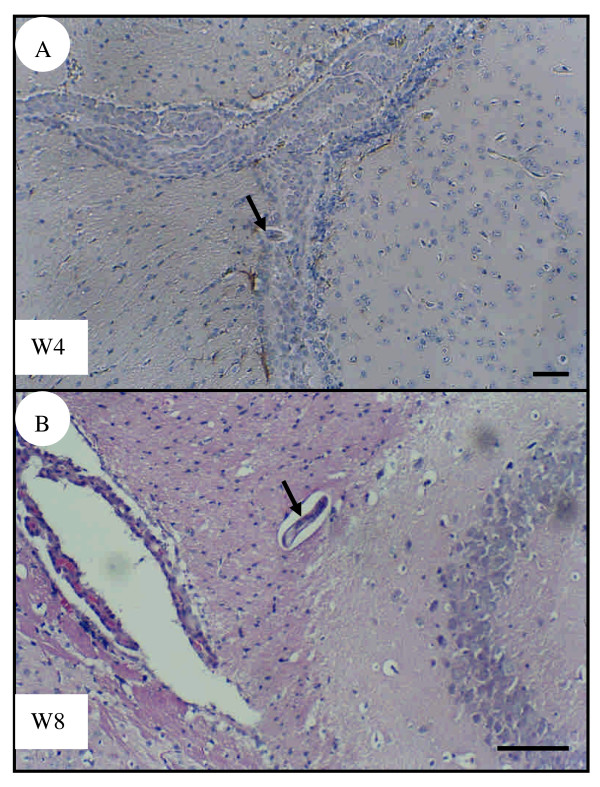
**Brain sections of *T. canis *infected mice.** Larvae (arrows) present in or in the vicinity of the choroid plexus, showing that there is no inflammatory cell infiltration in the brains of infected mice at 4 wpi (A) or 8 wpi (B). Tissue sections were stained with hematoxylin-eosin. Bar = 50 μm.

**Figure 4:**
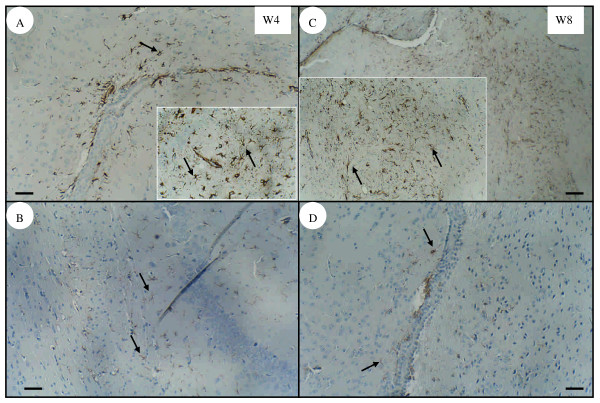
**Immunochemical staining of the glial fibrillary acidic protein (GFAP) in mouse brain.** Astrogliosis with apparent GFAP expression (arrow) was observed in the parenchyma near to the choroid plexus in the brains of infected mice at 4 wpi (A) or 8 wpi (C). However, weak GFAP expression was also detected in the brains of age-matched uninfected mice at 4 wpi (B) and 8 wpi (D). Bar = 50 μm. Inserts are higher magnifications of GFAP expression from the same panel.

No inflammatory cell infiltration was found in the experimental groups of infected mice at 3 dpi or 1 wpi (data not shown). Although larvae were found in or close to the choroid plexus, no leukocyte infiltration was seen in infected mice at 4 (Fig. 3A) or 8 wpi (Fig. 3B) as analyzed by H&E-stained sections. The immunohistochemical assessment showed that infected mice had many astrocytes with significant expression of GFAP in the cerebral parenchyma near the choroid plexus at 4 (Fig. 4A) and 8 wpi (Fig. 4C). However, weak GFAP expression was also detected in the age-matched control group of uninfected mice at either 4 (Fig. 4B) or 8 wpi (Fig. 4D).

### Induction of enhanced expressions of TGF-β1, S100B, GFAP, NF-L, tTG, AβPP, and -tau and p-tau proteins accompanied by impairment of the UPS during *T. canis *larval invasion of the mouse brain

**Figure 5:**
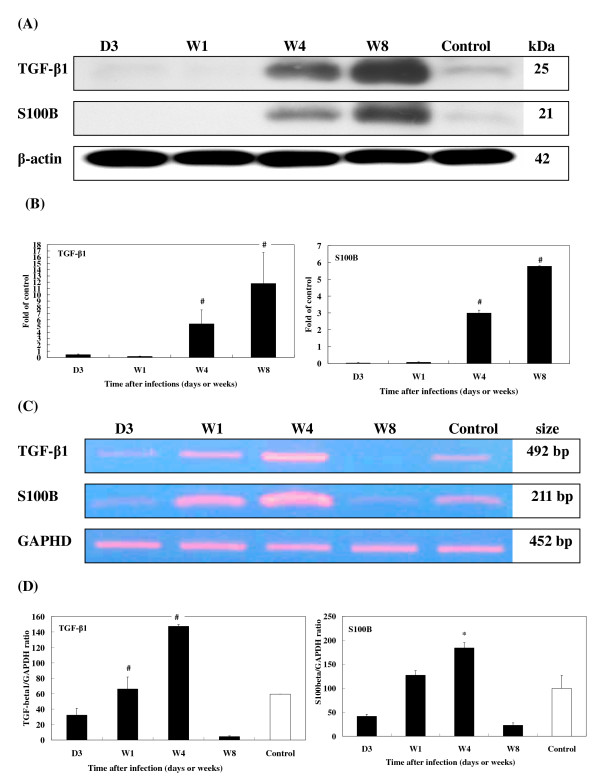
**Expression of TGF-β1 and S100B in the brains of *T. canis *infected mice.** (A) Protein levels of TGF-β1 and S100B in the brains of *T. canis *infected mice from 3 dpi to 8 wpi assessed by Western blotting. β-actin was used as a loading control. (B) Relative times were generated by comparing intensities between infected and uninfected mice. The error bars indicate the standard deviations and the superscript indicates significant differences from control; ^#^*P *< 0.001. (C) Expression levels of TGF-β1 and S100B in the brains of infected mice from 3 dpi to 8 wpi were examined by RT-PCR. GADPH was used as a reaction control. (D) The relative amounts of TGF-β1 and S100B mRNA were calculated based on the optical density relative to that of the GAPDH. The error bars indicate the S.D. and the superscripts represent significant differences from the control; *P < 0.05, ^#^*P *< 0.001. Three to eight mice per group were examined.

**Figure 6:**
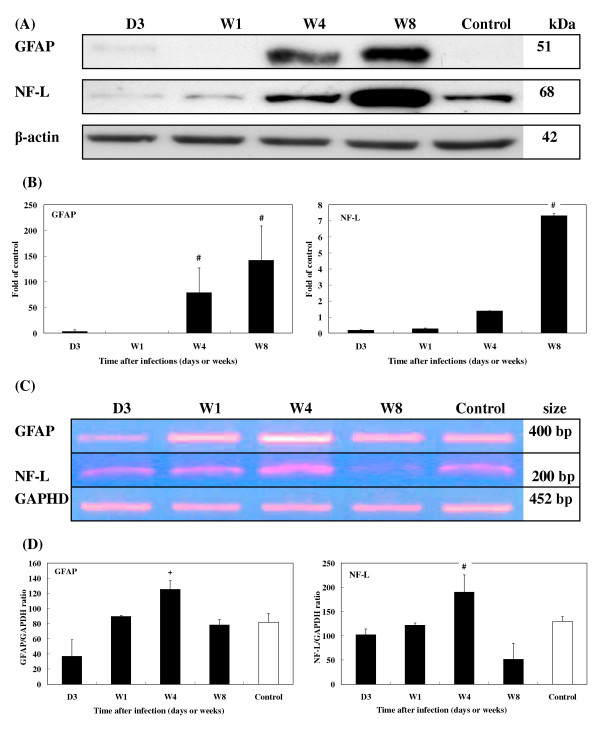
**Expression of GFAP and NF-L in the brains of *T. canis *infected mice.** (A) Protein levels of GFAP and NF-L in the brains of infected mice from 3 dpi to 8 wpi assessed by Western blotting. (B) Relative times were generated as described in Fig. 5B. The error bars indicate the S.D. and the superscript indicates significant differences from the control; ^#^*P *< 0.001. (C) Expression levels of GFAP and NF-L in the brains of infected mice from 3 dpi to 8 wpi were assessed by RT-PCR. (D) The relative amounts of GFAP and NF-L mRNA were calculated based on the optical density relative to that of the GAPDH. The error bars indicate the S.D. and the superscripts represent significant differences from the control; ^+^*P *< 0.01, ^#^*P *< 0.001. Three to eight mice per group were examined.

When *T. canis *larvae invaded the mouse brains, there were no changes in the level of either TGF-β1 or S100B cytokines at 1 wpi (Figs. 5A,B). However, the levels of both cytokines had significantly increased by 4 and 8 wpi. TGF-β1 increased by 5.3- and 11.8-fold and S100B by 3.0- and 5.8-fold, respectively (Fig. 5A, B). Expression of GFAP was slightly evident at 3 dpi at 3.0-fold, and levels were significantly higher at 4 (78.9-fold) and 8 wpi (142.0-fold) but not at 1 wpi (Fig. 6A, B). NF-L expression also greatly increased over time, with 1.4- and 7.5-fold increases at 4 and 8 wpi, respectively (Fig. 6A, B).

**Figure 7:**
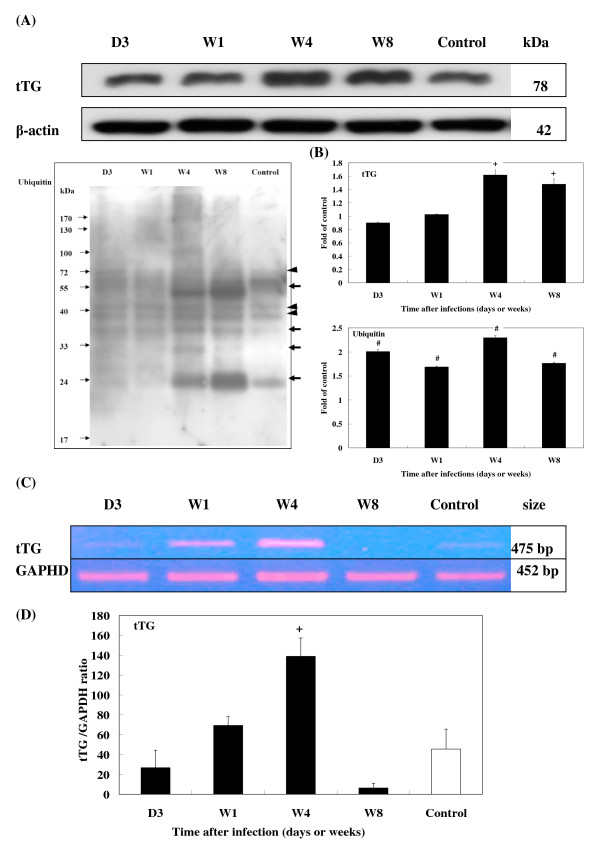
**Expression of tTG and ubiquitin in the brains of *T. canis *infected mice.** (A) Protein levels of tTG and ubiquitin (arrow head) and ubiquitylated protein (thick arrows) expression in the brains of infected mice from 3 dpi to 8 wpi were assessed by Western blotting. (B) Relative times were generated as described in Fig. 5B. The error bars indicate the S.D. and the superscripts represent significant differences to the control; ^+^*P *< 0.01, ^#^*P *< 0.001. (C) The expression levels of tTG in the brains of infected mice from 3 dpi to 8 wpi were assessed by RT-PCR. (D) The relative amount of tTG mRNA were calculated based on the optical density relative to that of the GAPDH. The error bars indicate the S.D. and the superscript represents significant differences from the control; ^+^*P *< 0.01. Three to eight mice per group were examined.

After infection, enzyme tTG levels had increased 1.6- and 1.5-fold by 4 and 8 wpi, respectively (Fig. 7A, B). Compared with levels found in uninfected controls, ubiquitin increased significantly from 3 to 8 wpi. Conjugated ubiquitins were more abundant at 4 and 8 wpi (Fig. 7A, B).

**Figure 8:**
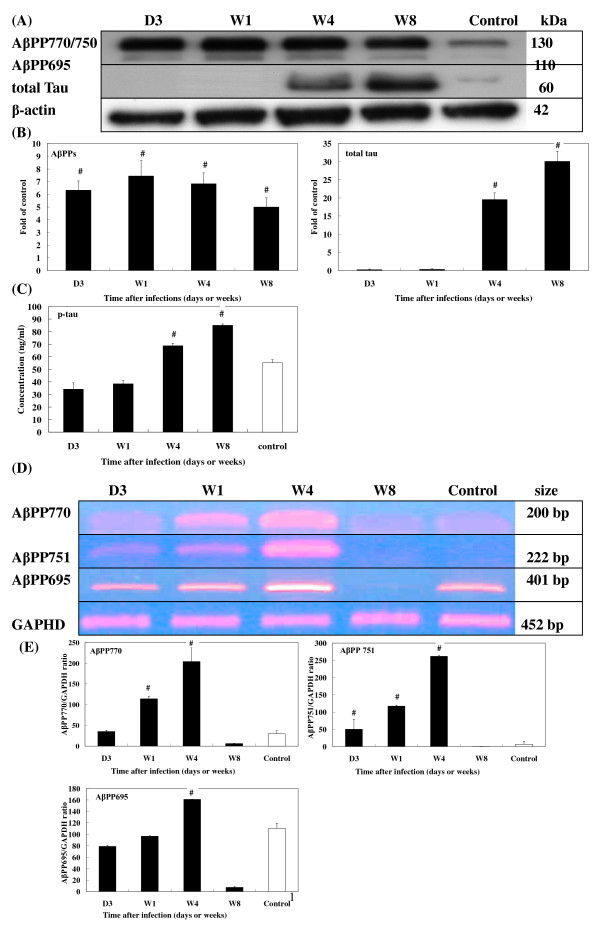
**Expression of AβPP and Tau in the brains of *T. canis *infected mice.** (A) Protein levels of AβPP and Tau in the brains of infected mice from 3 dpi to 8 wpi were assessed by Western blotting. (B) Relative times were generated as described in Fig. 5B. The error bars indicate the S.D. and the superscript represents significant differences to the control; ^#^*P *< 0.001. (C) Concentrations of phosphorylated Tau in the brains of infected mice from 3 dpi to 8 wpi were assessed by ELISA. The error bars indicate the S.D. and the superscript represents significant differences from the control; ^#^*P *< 0.001. (D) mRNA levels of AβPP expressions in the brains of infected mice from 3 dpi to 8 wpi were assessed by RT-PCR. (E) The relative amount of AβPP mRNA was calculated based on the optical density relative to that of the GAPDH. The error bars indicate the S.D. and the superscript represents significant differences from the control; ^#^*P *< 0.001. Three to eight mice per group were examined.

Levels of the neurotoxic protein, AβPP, progressively rose after infection with increases ranging 5.0~7.4-fold (Fig. 8A, B). Post-infection levels of t-tau also increased from 19.5-fold at 4 wpi to 30.6-fold at 8 wpi (Fig. 8A, B). Because expression of p-tau was undetectable by Western blot analysis, an ELISA kit was used to detect its expression. In brains from infected mice, p-tau was found to be 68.81 ± 2.06 ng/ml at 4 wpi (*P *< 0.001) and 85.11 ± 0.96 ng/ml at 8 wpi (*P *< 0.001), both significantly higher than those found in uninfected mice (55.25 ± 2.34 ng/ml) (Fig. 8C). However, no Aβ protein was detected (data not shown).

### Enhanced expressions of TGF-β1, S100B, GFAP, NF-L, tTG, and AβPPs mRNA during *T. canis *larval invasion of the mouse brain

RT-PCR revealed that the mRNA expression of the cytokines, TGF-β1 and S100B (Fig. 5C, D), glial proteins, GFAP and NF-L (Fig. 6C, D), and enzyme, tTG (Fig. 7C, D), coincided with their protein levels, with significantly elevated levels of mRNA seen at 1 and 4 wpi. By 8 wpi, all had steeply declined.

AβPP770, AβPP751, and AβPP695 levels significantly increased from 3 dpi to 4 wpi, followed by a significant decline at 8 wpi (Fig. 8D, E).

## Discussion

Although many cases of children and adults with CT clinically characterized by severe neurological disorders such as eosinophilic meningitis, encephalitis, myelitis, arachnoiditis, cerebral vasculitis, and dementia have not uncommonly been reported recently in developed countries, e.g., Austria [28], Belgium [29], France [30], Germany [31], Japan [21], and Switzerland [32] as well as in tropical developing countries, e.g., Brazil [33,34] and Turkey [35], the actual number of reported cases of cerebral infection with *T. canis *is still limited. In addition, brain involvement is likely too cryptic or not easily detected in humans with CT; thus, the underlying mechanism behind the pathogenesis and sequelae of subtle BI in CT has remained largely unclear. Because it is not easily detected, the actual number of cases of subtle BI caused by this tiny parasite has probably been underestimated. Worldwide, many asymptomatic cases of toxocariasis have been confirmed to be positive by serology [36], although in light or old infections, the dormant *Toxocara *larvae embedded in internal organs may be reactivated at any time and migrate again to the brain [37,38]. Despite the difficulties in detection, it is possible to use a murine model to study the underlying mechanism behind the pathogenesis of CT [19].

Although Hamilton et al. [19] recently indicated that BALB/c inbred strain mice are susceptible to *T. canis *infection and it seems that larvae remain in brains of BALB/c mice for a longer time compared to other strains of mice, using ICR mice as an animal model is still adequate to study CT. In fact, our previous study indicated that the larval recovery rate at a range of 2.9%~3.8% from the brain of ICR mice [39] was very close to that (4.4%) from the brain of outbred LACA mice inoculated with a low dose of 100 ova by Cox and Holland [40], who explored some effects on learning and memory of LACA mice with CT; in addition, Good et al. [41] also used CD1-ICR outbred mice to assess the larval distribution in the brain and found that the dose and brain region were significant factors. It is noteworthy that Cox and Holland [40] also, however, indicated that particularly in mice inoculated with a low dose of *T. canis *eggs, the low burden of *T. canis *larvae (an average larval burden of 6 larvae/brain; range, 0~15 larvae/brain) appearing in the brain and causing changes in murine behavior is likely to more realistically reflect the situation in humans and wild rodents with toxocariasis. Our present results of a mean larval burden per brain of 2.9 ± 3.0 larvae in ICR mice are close to that reported by Cox and Holland [40]. In addition, Dubinsky et al. [42] examined brains of 476 small mammals from Slovakia and found the numbers of larvae to range from 1 to 13 per brain, with a peak average being 4.2 ± 4.1 larvae/brain in animals collected from a suburban location. The low numbers of larvae harbored by brains of infected mice described in this paper are also very similar to those described by Dubinsky and colleagues. In answering why variations in larva recovery rates exist in individual brains, Kayes and Oakes [43] indicated that fluctuations in larval number might reflect the ability of larvae to migrate into and out of sampled tissues including the brain over time.

We found that *T. canis *larvae increasingly migrated to the brains over time, although our H&E staining showed no leukocyte infiltration or pathological changes in areas near the choroid plexus, the site of the *T. canis *invasion. The reason that inflammatory cell infiltration was not seen in the injured brain caused by *T. canis *larvae might be that the *T. canis *larvae mimic host tissue antigenic components and escape immune recognition or perhaps some mechanisms in the brain diminish inflammation in order to protect it from severe injuries caused by inflammatory storms [18]. However, there was cerebral injury, as evidenced by the apparent astrogliosis with enhanced expression of GFAP extending around injured areas where the increases appeared to be correlated with the number of larvae migrating into the brain over time, as seen from 4 to 8 wpi. These increases may be a response to help protect the brain from damage in experimental CT, as many studies have indicated that reactive astrocytes rapidly increase by producing massive GFAP in response to brain injury. They can extend far from the actual site of damage, thereby forming a glial scar that can act as a physical barrier between damaged and healthy cells and help reestablish an intact blood-brain barrier (BBB) [11,44]. Modulation of astrogliosis with enhanced GFAP expression which is responsible for brain injury in experimental CT was proposed to occur by TGF-β1 since some evidence has revealed that astrocytes treated with TGF-β1 exhibit a rapid and dose-dependent increase in the expression of GFAP mRNA, and this increase is translated into a change in the steady-state level of GFAP protein [45,46].

Although TGF-β1 is often considered an anti-inflammatory molecule, we still propose that enhanced TGF-β1 expression may play a certain role in promoting inflammation in acute brain injury associated with experimental CT. Substantial evidence indicates that injection of an antiserum directed against TGF-β1 reduces inflammation in the CNS after traumatic injury [47,48], and astroglial overproduction of TGF-β1 enhances inflammatory CNS disease in transgenic mice [49]. In addition, Lesne et al. [50], working with murine and human astrocyte cultures cultured with TGF-β1, suggested that TGF-β1 can be harmful because it promotes perivascular inflammation, interactions with and increased production of AβPP, and subsequent Aβ generation. Moreover, TGF-β1 has also been implicated in the pathogenesis of neurological diseases through its modulation of massive extracellular matrix (ECM) production and has been identified in amyloid plaques of AD brains. ECM proteins appear to play a central role in the deposition of Aβ [51].

Elevated expression of AβPP should have deleterious effects on the acute brain injury of experimental CT. This postulation was confirmed by AβPP being involved in cerebral falciparum malaria (CFM) which causes disruption of axonal transport leading to neurological dysfunction as supported by the frequency and extent of AβPP staining being more severe in patients with than in those without CFM [52]. It is noteworthy that an increase of isoform AβPP751/770 protein levels but a decline in the main neuronal isoform AβPP695 protein level was observed to progress over time in experimental CT, changes that are very similar to those seen in the rat brain after TBI [53]. AβPP transgenic mice overexpressing human AβPP695 had an accentuation of presynaptic terminal loss with no sprouting re action in the outer molecular layer of the hippocampus following perforant pathway transaction, compared to non-transgenic controls, which had a normal reinnervation pattern [54]. Moreover, brains of human AβPP751-expressing transgenic mice were shown to be labeled by antibodies directed against Aβ, possibly consistent with early AD-type CNS alterations [55].

Enhanced expression of AβPP appears to make possible AβPP intraneuronal processing through which Aβ peptides, which are potentially detrimental to the brain, are produced [14]. Although we found increased protein levels of AβPP, we found no Aβ peptides in the brains used in this study examining experimental CT. The absence of these peptides might be partly due to larvae not having resided in the brains for a sufficiently long period for deposition of Aβ to become visible, or perhaps because Aβ expression is barely detectable in normal non-transgenic mice. Previous studies reported Aβ expression to be undetectable in standard rodent models of focal brain trauma and suggested that this might be explained by differences in rodent and human metabolism of Aβ [56].

The role of tTG induction in the neural response to injury in experimental CT is not clear. Some studies found upregulation of tTG after TBI [6]. tTG expression has also been implicated in the cellular pathogenesis of AD as evidenced by tTG-specific immunoreactivity observed in neuritic plaques and amyloid cores, and tau was found to readily be cross-linked by tTG [57,58]. In the present study, we observed 20~30-fold increases in total protein levels of tau at 4 and 8 wpi in experimental CT, possibly a consequence of nerve cell damage. Similar findings were reported in AD, Creutzfeldt-Jakob disease, Guillain-Barré syndrome, TBI, and severe cerebral falciparum malaria [59,60]. It is noteworthy that we also found greater levels of hyperphosphorylated tau protein in the brains of infected mice than in those of uninfected mice at 4 and 8 wpi. Nevertheless, due to the lack of direct evidence of neurofibrillary tangle (NFT) deposition in the brain, we do not know whether hyperphosphorylated tau plays a role in promoting the development of an AD-like syndrome in experimental CT. It was suggested that hyperphosphorylated tau, probably initiated by amyloid deposition, might cause NFT in AD [15].

Similarly, it was suggested that elevated S100B levels are involved in the development of Down's syndrome and AD [61]; its expression was also correlated with the density of dystrophic neuritis with overexpressed AβPP [62]. Recent evidence indicates that high levels of S100 B in CSF increase the risk of repeated seizures in children with severe *P. falciparum *infection due to axonal injury [60]. It was found that excess accumulation of NF-L has a detrimental effect on nerve cells resulting from NF-L inclusions in axons mechanically blocking the transportation of particles through axons, which eventually leads to neuronal death and to abnormalities in and degeneration of motor neurons [63]. Excess accumulation of NF-L was also observed in several neurodegenerative diseases, e.g., amyotrophic lateral sclerosis, AD, Lewy bodies in Parkinson's disease, Charcot-Marie-Tooth disease, and giant axonal neuropathy [12].

Interestingly, while mRNA expressions of TGF-β1, S100B, GFAP, N F-L, tTG, and AβPP markedly declined in brains of the experimental CT mice at 8 wpi, their corresponding proteins remained abundantly present at that time. One possibility for this might be that transcription of those BIABs, which began from 4 wpi onwards, had ceased by 8 wpi. The fact that these proteins were still detectable at 8 wpi may be explained by the time lag in translating mRNAs or by the stability and persistence of the proteins. We explored the possibility that some protein degradation systems, e.g., the UPS, were disabled or impaired in brains of the experimental CT mice between 4 and 8 wpi. Ubiquitin conjugates as well as elevated ubiquitin levels in our study were found to be increased in brains of experimental CT mice, indicating impairment of the UPS's processing of excess unwanted proteins [16]. Several studies proposed that intracellular deposition of ubiquitylated proteins and increased ubiquitin contents are a prominent cytopathological feature of AD [64,65]. However, further studies are needed to clarify the link between enhanced BIAB expression and UPS impairment. Why are levels of TGF-β1, S100B, and NF-L of infected mice at 3 dpi or 1 wpi lower than those of uninfected mice, or why are there discrepancies between biomarker protein, TGF-β1, S100B, and NF-L mRNA expressions at 8 wpi (Figs. 5A, 6A)? It is rather difficult to explain why mRNA or protein expressions of those biomarkers in infected mice are weaker than those in uninfected mice in early infection, e.g., at 3 dpi or late infection, e.g., at 8 wpi, since in early infection, no or few larvae have invaded the brain, while more larvae have migrated into the brain in late infection. We, however, propose that in early or late infection some unclear mechanisms, e.g., cytokines or stress may affect the stability of TGF-β1, S100B, and NF-L mRNA, which attenuates their expressions in the brains of infected mice; however, this postulation and their corresponding pathophysiological functions during those periods of infection should be further tested.

*Toxocara canis *larvae have been shown to be non-randomly distributed within the brain, with the telencephalon and cerebellum being their preferred sites of accumulation after at least 1 wpi, as evidenced by studies using different strains of mice inoculated with a single dose of *T. canis *eggs [66-68]. Considering that these brain areas are associated with memory and coordination, cerebral infection with *T. canis *should have potent impacts on different aspects of murine behavior. Several studies have reported varying degrees of behavior changes, and others have reported some level of memory impairment in either inbred or outbred murine hosts infected with *T. canis *[40,41,19]. Although several studies have indicated that abnormal behavioral changes may be related to the larval burden [41,19], there is no evidence that mechanical damage caused by migrating larvae in the brain tissue is the main cause of symptoms in experimental CT [69]. Since several studies on other parasitic infections of the CNS have demonstrated that cytokines produced in response to infection are often responsible for the induction of pathologies and neurodegeneration [70-72], and in addition, it was recently proposed that immune responses elicited by cytokines, interleukin-5, interferon-γ, and inducible nitric oxide synthase in the brain may be other factors influencing abnormal behavior changes or pathology in mice with CT [20], we propose that enhanced BIAB expressions in brains of mice with *T. canis *infection in the present study may provide more insights to better understand the pathogenesis of CT and its links with the possible development of neurodegeneration.

## Conclusion

To the best of our knowledge, this is the first study to provide clear evidence of the concomitant presence of enhanced BIAB expression and UPS impairment during *T. canis *larval invasion of the brain. Although further studies regarding the relationship between the expressions of various BIABs and behavior disorders in experimental CT are required, results of the present study and those of the others cited in this paper suggest the possibility that cerebral infection by *T. canis *can have deleterious consequences and may increase the risk that CT will develop into neurodegenerative-like disease such as AD. This cannot be completely excluded because neurodegeneration is associated with the emergence of AβPP and phosphorylated tau in the brains of experimental CT mice.

## Abbreviations

TGF-β1: transforming growth factor β1; GFAP: glial fibrillary acidic protein; NF-L: neurofilament light chain; tTGs: tissue transglutaminases; AβPPs: β-amyloid precursor proteins; UPS: ubiquitin-proteasome system; BIAB: brain injury associated biomarkers; AD: Alzheimer's disease; TBI: traumatic BI; CT: cerebral toxocariasis.

## Competing interests

The authors declare that they have no competing interests.

## Authors' contributions

C–KF conceived of the study, participated in the design of the study, coordination, drafted the manuscript, and performed the statistical analysis. C–KF, C–WL and W–LC carried out the molecular studies, pathological studies, and drafted the manuscript. T–CK, K–ES, and D–DJ carried out the larva recovery study, immunoassays, and gave writing suggestions. Y–HL carried out the pathological studies. All authors read and approved the final manuscript.

## Pre-publication history

The pre-publication history for this paper can be accessed here:


